# A Case of Separate *os tarsale primum* in a Horse

**DOI:** 10.3390/vetsci13060582

**Published:** 2026-06-15

**Authors:** Diyana Vladova, Dimitar Kostov, Hristo Hristov, Nikolay Goranov, Tihomir Dinev, Avche Lioteva-Dineva, Kamelia Stamatova-Yovcheva

**Affiliations:** 1 Department of Veterinary Anatomy, Histology and Embryology, Faculty of Veterinary Medicine, Trakia University, 6000 Stara Zagora, Bulgaria; d_kostov62@abv.bg (D.K.); hristo.hristov@trakia-uni.bg (H.H.); tihomir.dinev@trakia-uni.bg (T.D.); avche.lyoteva@trakia-uni.bg (A.L.-D.); 2 Department of Diagnostic Imaging, University Veterinary Hospital, Trakia University, 6000 Stara Zagora, Bulgaria; nikolay.goranov@trakia-uni.bg

**Keywords:** *os tarsale primum*, *articulatio tarsi*, horse

## Abstract

Anatomical variation in the equine tarsus may involve either fusion of the *os tarsale primum* with the *os tarsale secundum* or the presence of a separate *os tarsale primum*. The distal row of tarsal bones is therefore of particular anatomical interest, as an independent first tarsal bone has been only sporadically documented in previous studies. In this study, twelve equine tarsal joints were examined using macroscopic and radiographic methods. In eleven specimens, six tarsal bones were identified, whereas in one specimen (1/12, 8.3%), seven tarsal bones were observed due to the presence of an independent *os tarsale primum* with an irregular cuboid shape. Radiographic examination confirmed and complemented the macroscopic findings. These results document the morphological characteristics of the distal tarsal region in the examined specimens and may support further anatomical and diagnostic studies of the equine tarsal joint.

## Simple Summary

The equine tarsal (hock) joint may show anatomical variations, including those involving *os tarsale primum*. In this study, twelve equine tarsal joints were examined using macroscopic and radiographic methods. In most cases, six tarsal bones were identified, whereas in one specimen an additional bone was observed, resulting in a configuration of seven bones. These findings contribute to the understanding of anatomical variations and their interpretation in diagnostic imaging.

## 1. Introduction

The equine tarsal joint has a complex anatomical structure and intricate arrangement of its osseous components, which creates challenges for both anatomical description and diagnostic imaging. Detailed knowledge of tarsal osteoanatomy is essential for accurate diagnosis and appropriate clinical decision-making [1,2]. This is particularly relevant for the distal row of the tarsal bones, where variations in fusion or independent presentation of individual elements have been reported.

Embryological studies indicate that ossification of the tarsal bones begins during the fetal period and may influence the final morphological configuration of the tarsus [3]. These developmental processes suggest that variations in the degree of fusion between *os tarsale I* and *os tarsale II* may be due to anatomical variants rather than pathological conditions.

Classically, *os tarsale I* (t I) and *os tarsale II* (t II) are described as a single fused structure, referred to as *os cuneiforme mediale* or *os cuneiforme mediointermedium* [4,5,6,7,8,9]. This synostosis reduces the number of tarsal bones to six, with three bones forming the distal row. The *os cuneiforme mediale* is typically characterized by a medioplantar position, a flattened proximodistal shape, and articular facets for *os tarsi centrale* and *os metatarsale II* [7]. However, several studies have reported variability in this fusion pattern, ranging from complete synostosis to partial or independent presentation of t I and t II [10,11,12,13].

Advanced imaging modalities complement classical anatomical descriptions and allow more precise evaluation of skeletal structures. Computed tomography has demonstrated a high correlation with cross-sectional anatomy, supporting the identification of anatomical variations [10]. In clinical practice, radiography remains the primary imaging modality for evaluation of the tarsal region, mainly for detection of fractures and traumatic lesions [14]. In this context, recognition of an independent *os tarsale I* is important, as it may be misinterpreted as a fracture line or bone fragmentation.

Furthermore, specific configurations of the tarsal bones, such as wedging of the central and third tarsal bones, have been reported as a potential predisposing factor for degenerative joint disease [15,16].

The aim of the present study was to describe the morphological characteristics of a separately developed *os tarsale primum* in the equine distal tarsal row and to evaluate its diagnostic and clinical relevance based on anatomical and radiographic findings.

## 2. Materials and Methods

Twelve anatomical specimens, each representing an individual equine tarsal joint, were available in the teaching anatomical collection of the Department of Veterinary Anatomy, Histology and Embryology, Faculty of Veterinary Medicine, Trakia University. The specimens were obtained from animals used for educational purposes and were prepared using standard veterinary anatomical dissection techniques. Preparation included systematic removal of soft tissues, mechanical cleaning of osseous structures, and subsequent preservation for instructional use. No chemical fixation or additional experimental processing was applied.

All twelve specimens were subjected to macroscopic anatomical examination. The specimens were selected from the collection without prior case selection; no information regarding age, breed, or sex was available. The sample included six left and six right tarsal joints.

During routine examination of the teaching collection, one specimen was identified as exhibiting a separate *os tarsale primum*. This specimen was subsequently subjected to detailed macroscopic and radiographic evaluation and is presented in this report. Radiographic examination was performed with an X-ray system (Philips Healthcare, Hamburg, Germany), and the images were processed with direct radiography (Philips Bucky Diagnost C 94, Hamburg, Germany). The exposure parameters were 55 kV and 10 mAs. The focal distance was 85 cm, and the specimens were imaged in mediolateral and dorsoplantar projections. The specimens were manually positioned and stabilized in the desired anatomical orientation during radiographic acquisition.

The radiographic examination was performed at the Radiology unit of the University Veterinary Hospital, Faculty of Veterinary Medicine, Trakia University. To ensure consistency, all radiographic images were acquired and interpreted by the same experienced veterinary radiologist.

## 3. Results

### 3.1. Tarsal Bones (Macroscopic Findings)

The tarsal bones of the examined anatomical specimens were short and arranged in three rows: proximal (crural), intermediate (central), and distal (metatarsal). The proximal row was represented by the talus and calcaneus, and the intermediate row by the *os tarsi centrale*.

In eleven specimens (11/12, 91.7%), the distal row consisted of six tarsal bones due to fusion of *os tarsale primum* and os *tarsale secundum*, consistent with the typical anatomical configuration of the equine tarsus.

**Figure 1:**
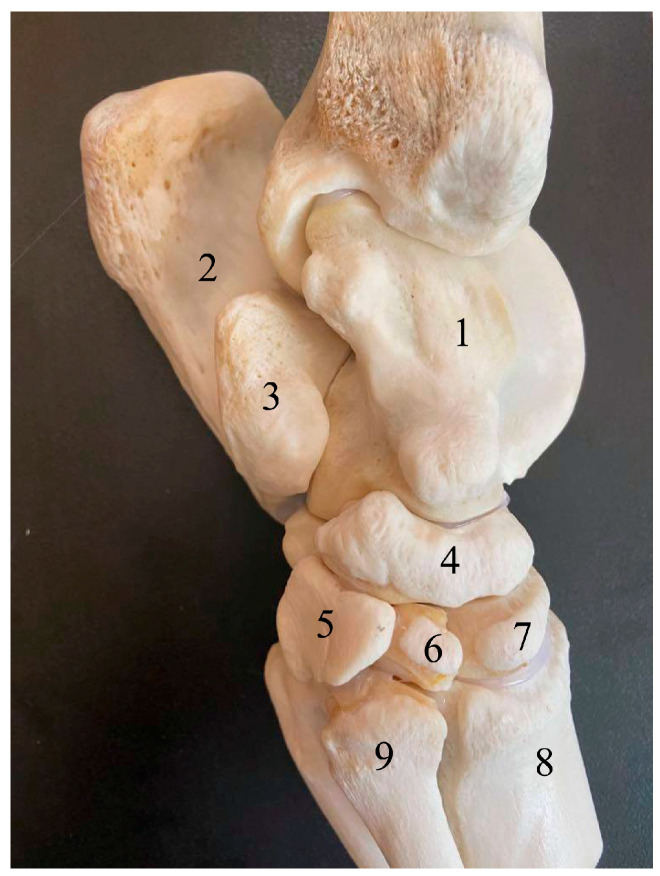
Medial view of a skeletal anatomical preparation of the left equine tarsal region. (1) *talus*; (2) *calcaneus*; (3) *sustentaculum tali*; (4) *os tarsi centrale*; (5) *os tarsale primum*; (6) *os tarsale secundum*; (7) *os tarsale tertium*; (8) *os metatarsale tertium*; (9) *os metatarsale secundum*.

In one specimen (1/12, 8.3%), a well-defined *os tarsale primum* of irregular cuboid shape, larger than the *os tarsale secundum*, was identified in the distal row. In this specimen, a total of seven tarsal bones were included, and no fusion between *os tarsale primum* and *os tarsale secundum* was observed (Figure 1).

**Figure 2:**
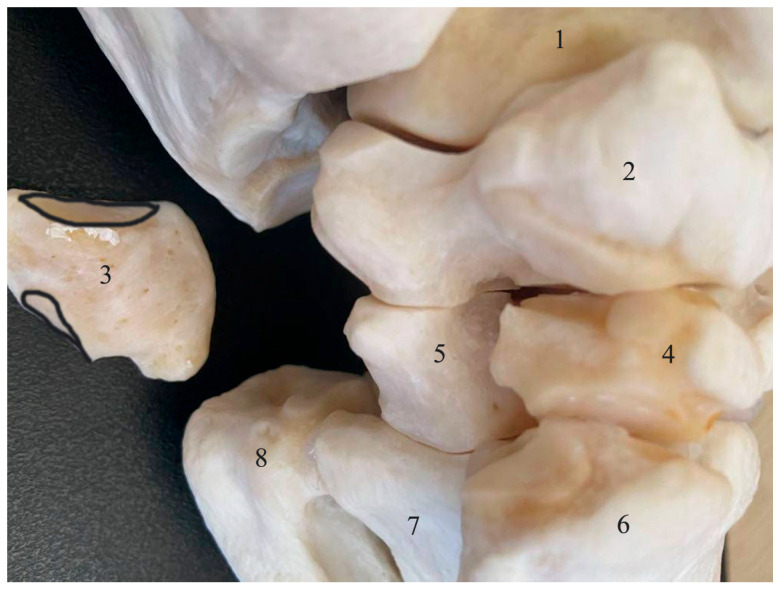
Medioplantar view of a skeletal anatomical preparation of the left equine tarsal region (*os tarsale primum* is dissected and clearly separated, and its articular facets are highlighted). (1) *Talus*; (2) *os tarsi centrale*; (3) *os tarsale primum*; (4) *os tarsale secundum*; (5) *os tarsale tertium*; (6) *os metatarsale secundum*; (7) *os metatarsale tertium*; (8) *os metatarsale quartum*.

After separation of *os tarsale primum*, the first tarsal bone in this specimen, two well-defined articular facets were observed proximally and distally, corresponding to articulation with the *os tarsi centrale* and the second and third metatarsal bones (Figure 2).

### 3.2. Radiographic Findings

Radiographic examination of the specimen showing the anatomical variation confirmed and complemented the macroscopic findings.

**Figure 3:**
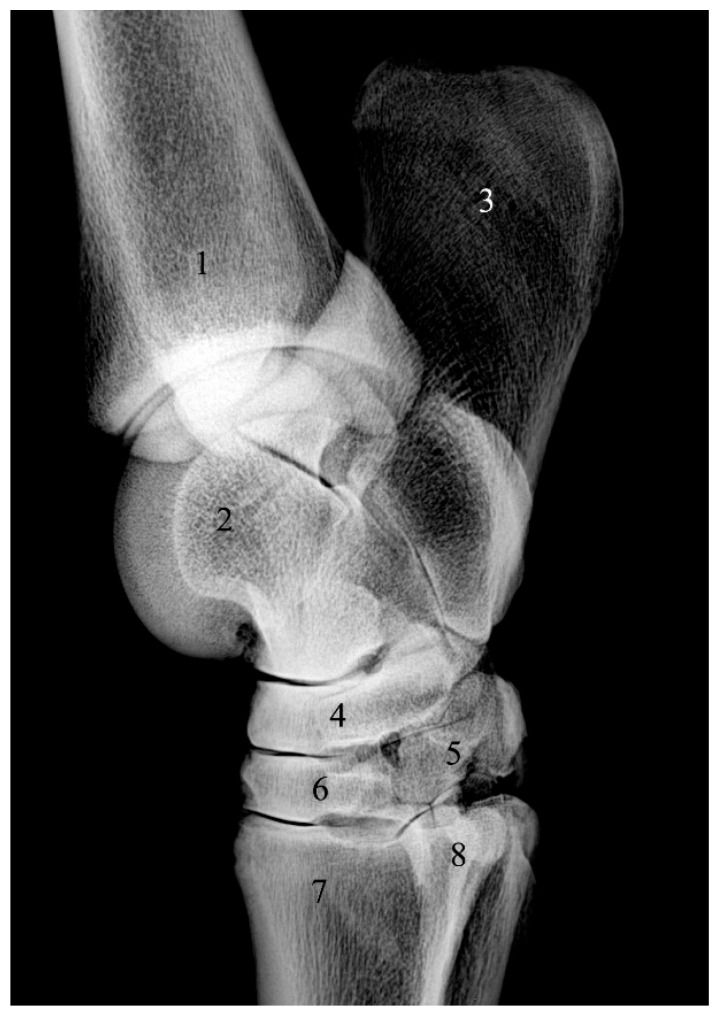
Radiographic image of the right equine tarsal region. Mediolateral projection. (1) *Tibia*; (2) *talus*; (3) *calcaneus*; (4) *os tarsi centrale*; (5) *os tarsale primum et secundum*; (6) *os tarsale tertium*; (7) *os metatarsale tertium*; (8) *os metatarsale secundum*.

**Figure 4:**
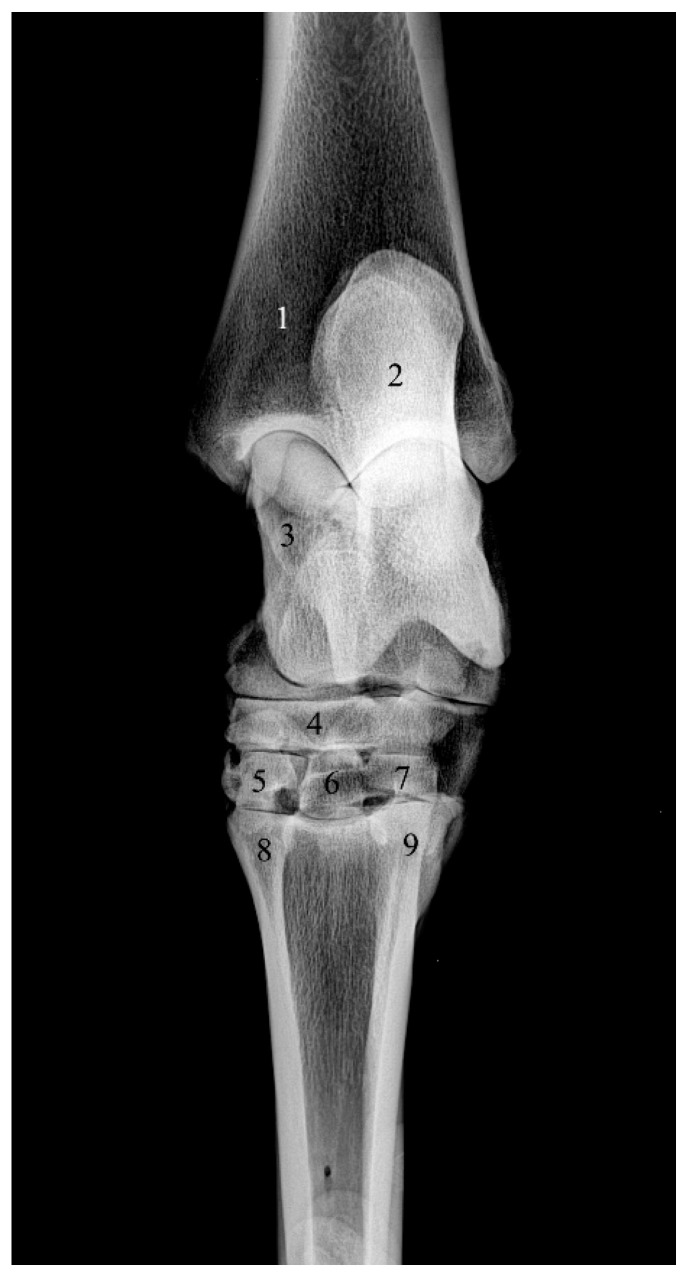
Radiographic image of the right equine tarsal region. Dorsoplantar projection. (1) *Tibia*; (2) *calcaneus*; (3) *talus*; (4) *os tarsi centrale*; (5) *os tarsale primum et secundum*; (6) *os tarsale tertium*; (7) *os tarsale quartum*; (8) *os metatarsale secundum*; (9) *os metatarsale quartum*.

In eleven specimens, radiographs demonstrated the typical configuration of six tarsal bones, with fusion of *os tarsale I* and *os tarsale II* (Figure 3 and Figure 4). In these specimens, the fused *os cuneiforme mediale* articulated proximally with the *os tarsi centrale* and distally with the second and third metatarsal bones.

**Figure 5:**
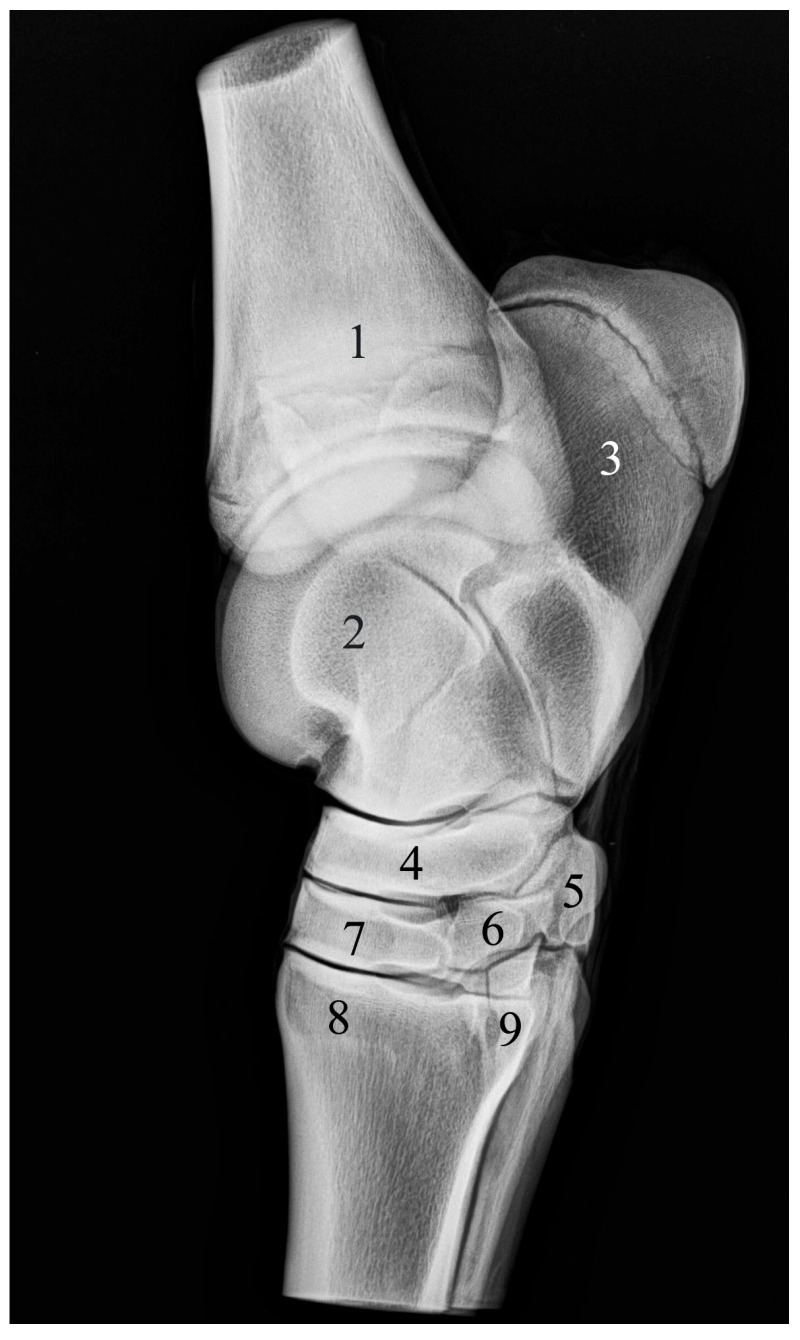
Radiographic image of the right equine tarsal region. Mediolateral projection. (1) *Tibia*; (2) *talus*; (3) *calcaneus*; (4) *os tarsi centrale*; (5) *os tarsale primum*; (6) *os tarsale secundum*; (7) *os tarsale tertium*; (8) *os metatarsale tertium*; (9) *os metatarsale secundum*.

In the single specimen (1/12), mediolateral projection revealed a well-defined radiopaque structure corresponding to the *os tarsale primum*, in contact with the *os tarsi centrale*, *os tarsale tertium*, and the second and third metatarsal bones. The structure was clearly separated from the *os tarsale secundum*, with no evidence of synostosis or fusion (Figure 5).

**Figure 6:**
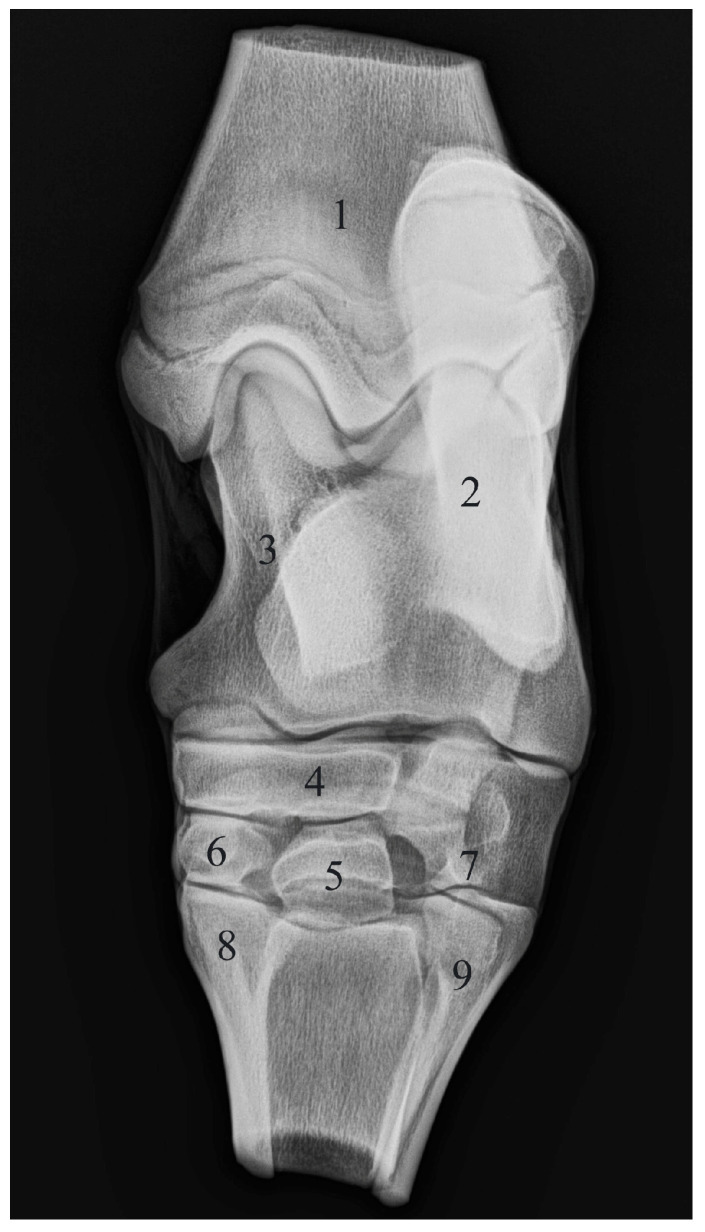
Radiographic image of the right equine tarsal region. Dorsoplantar projection. (1) *Tibia*; (2) *calcaneus*; (3) *talus*; (4) *os tarsi centrale*; (5) *os tarsale primum* superimpossed over *os tarsale tertium*; (6) *os tarsale secundum*; (7) *os tarsale quartum*; (8) *os metatarsale secundum*; (9) *os metatarsale quartum*.

In the dorsoplantar projection of the same specimen, the three-row arrangement of the tarsal bones was confirmed. The proximal row consisted of the *talus* and *calcaneus*, and the intermediate row was represented by the *os tarsi centrale*. The *os tarsale primum* was identified in the distal row as a distinct radiopaque structure with a well-defined cortical margin and a radiolucent trabecular bone, in contact with the *os tarsi centrale*, *os tarsale secundum*, *os tarsale tertium*, and the second and third metatarsal bones (Figure 6).

## 4. Discussion

### 4.1. Key Findings

The present findings describe an unusual anatomical configuration of the distal tarsal bones in the horse, characterized by the presence of a distinct *os tarsale primum* and an overall arrangement of seven tarsal bones. This configuration was consistently identified through combined macroscopic and radiographic evaluation, providing both complementary anatomical and imaging evidence of this variation.

### 4.2. Anatomical Context and Variability

The observed configuration differs from the classical description of a fused *os cuneiforme mediale* (*os cuneiforme mediointermedium*) as reported in standard anatomical literature [4,5,6,7,8,9]. However, previous studies have documented variability in the degree of fusion and morphological presentation of the distal tarsal bones [10,11,12,13], suggesting that different patterns of ossification and fusion may occur within this anatomical region. The present observation provides additional evidence supporting the existence of such anatomical variability described in earlier anatomical and developmental studies.

### 4.3. Clinical and Diagnostic Implications

The identification of an independent *os tarsale primum* may be relevant for the interpretation of equine tarsal radiographs as such a structure could potentially be misinterpreted as a fracture line or bone fragment during routine diagnostic evaluation [14]. The absence of synostosis between *os tarsale primum* and *os tarsale secundum* in the examined specimen indicates a morphological variation rather than a pathological condition [3]. Recognition of this anatomical variation, which represents a single individual anatomical finding, is important for improving diagnostic accuracy and supporting appropriate differential diagnosis in the tarsal region [1,2,3]. In addition, awareness of such variability may contribute to more precise interpretation of degenerative or traumatic changes in the equine hock region [15,16].

### 4.4. Limitations

This study is limited by the examination of a single specimen derived from a collection of teaching materials. As only one out of twelve examined tarsal specimens exhibited this configuration, this represents an isolated case within the examined sample, and no population-level conclusions can be drawn regarding its frequency or distribution. Furthermore, the lack of complete metadata (including age, breed, and sex) for the examined specimens limits broader comparison with previously reported anatomical datasets.

### 4.5. Future Directions

Further investigations including a larger number of specimens, well-documented population data, and additional imaging modalities (e.g., computed tomography and oblique radiographic projections) are required to better characterize the frequency, developmental basis, and potential clinical relevance of this anatomical variation.

## 5. Conclusions

The present study documents an anatomical variation observed in a single equine tarsal specimen, representing an isolated case within the examined collection, expressed in a well-shaped and well-developed *os tarsale primum*, which was in anatomical contact with *os tarsi centrale*, *os tarsale secundum*, *os tarsale tertium* and mt II, and mt III. These features may be relevant for the anatomical understanding and the interpretation of diagnostic imaging of the equine tarsal region, particularly in cases where there is potential for misinterpretation in suspected trauma.

## Data Availability

The original contributions presented in this study are included in the article. Further inquiries can be directed to the corresponding authors.

## Abbreviations

t I	*os tarsale I*
t II	*os tarsale II*
mt II	*os metatarsale secundum*
mt III	*os metatarsale tertium*
